# High‐Frequency Ultrasound Assessment of Skin Thickness Following Lipofilling in Facial Sclerosing Dermatoses: A Case Series

**DOI:** 10.1111/jocd.70229

**Published:** 2025-05-22

**Authors:** Sofia Gomez‐Martinez, Irene Fuertes, Ada Ferrer Fuertes, Francisco Javier Cuesta Gonzalez, Carlos Martí Pagés, Gema M. Lledó‐Ibáñez, Gerard Espinosa, Monica Quintana‐Codina, Maribel Iglesias Sancho, M. Dolores Cembrano, Xavier Bosch‐Amate, José‐Manuel Mascaró, Priscila Giavedoni

**Affiliations:** ^1^ Dermatology Department Hospital Clínic de Barcelona, University of Barcelona Barcelona Spain; ^2^ Maxillofacial Surgery Department Hospital Clínic de Barcelona Barcelona Spain; ^3^ Autoimmune Systemic Diseases Department Barcelona Spain; ^4^ Dermatology Department Hospital Universitari Sagrat Cor, Grupo Quironsalud Barcelona Spain; ^5^ Dermatology Department Fundación Jiménez‐Díaz Madrid Spain

**Keywords:** autoimmune, lipoatrophy, lipolifting, ultrasound, ultrasound measurements

To the Editor,

Facial sclerosing inflammatory dermatoses (FSD) are characterized by irreversible dermal and subcutaneous tissue loss, leading to cosmetic and functional impairment [1]. Autologous fat grafting or lipofilling improves contour irregularities in facial morphea by restoring volume and promoting tissue remodeling through adipose‐derived stem cell activity [2, 3]. High‐frequency cutaneous ultrasound (HFU) is a non‐invasive and accessible tool that objectively asseses dermal and subcutaneous tissue thickness and disease activity through echogenicity and vascularity [4, 5]. We present a multicenter retrospective series of eleven patients with facial atrophy due to progressive facial hemiatrophy (*n* = 6), morphea “en coup de sabre” (*n* = 2), lupus panniculitis (*n* = 2), and dermatomyositis (*n* = 1) who underwent lipofilling according to the Coleman technique (median age 44.5 years, IQR 30–52, 81% female; Table 1). The number of lipofilling sessions ranged from one to five, based on clinical evaluation of atrophy. Informed consent was obtained from all participants. HFU was performed at baseline and at a median of 3.1 months after the final lipofilling in all patients, with an additional evaluation at 4.2 months after the first session in nine patients. Ultrasound measurements, acquired with a MyLab Class C Esaote instrument (Genova, Italy) and a 18–22 MHz probe, included thickness from the epidermis to the bone and Doppler activity (Figure 1b,d). Tissue loss was expressed in millimeters and as a percentage relative to the unaffected side, considered 100%. In the dermatomyositis case with bilateral loss, final thickness after the last lipofilling was set at 100% due to complete aesthetic restoration (Figure 1a,c). Statistical analysis was performed using Wilcoxon signed‐rank tests in R (version 4.4.0, R Core Team, 2023). The median tissue thickness on the affected side increased from 50% of the unaffected side at baseline (IQR 47–57) to 83% after the final lipofilling session (IQR 74–100; *p* < 0.01). In the subgroup of patients with HFU available after the first procedure, median thickness of the affected side increased from 82% after the first lipofilling (IQR 77–94) to 100% after the last session (IQR 99–100; *p* = 0.01). The cheek showed the highest average thickness increase (5.04 mm, *p* < 0.01), followed by the malar area (3.4 mm, *p* = 0.02) and forehead (2.34 mm, *p* = 0.03), while the increase in the chin (2.32 mm, *p* = 0.06) was not significant. HFU revealed hypoechogenicity or mixed echogenicity in the subcutaneous layer of the treated areas, with poorly defined margins and a loss of clear dermal–subcutaneous differentiation. We did not observe increased vascularization, signs of fat necrosis or cyst formation in any patients. To our knowledge, this is the first study to evaluate lipofilling outcomes in FSD using HFU. In this procedure, facial morphea has been associated with significantly reduced fat graft retention compared to healthy skin, primarily due to prior corticosteroid treatment and the presence of a local inflammatory microenvironment [3]. Despite these challenges, a statistically significant increase in tissue thickness was observed after the first lipofilling session and continued to improve with subsequent procedures, suggesting limited resorption over time. Fat graft survival appears to vary by anatomical site, with the forehead yielding better cosmetic outcomes than the nose or chin—which aligns with our results [3]. No complications were reported, supporting both the efficacy and safety of the technique. Follow‐up in lipofilling is limited as it mainly relies on subjective aesthetic evaluations. HFU may represent an essential tool to guide the timing of additional sessions by reliably monitoring tissue thickness and confirming the absence of inflammation. Limitations of the study include the small sample size, the absence of a control group, retrospective design, and the lack of patient‐reported outcome scores. In conclusion, HFU is a valuable tool for objective assessment of lipofilling efficacy in FSD.

**TABLE 1:** Patient characteristics and evolution of skin thickness on the atrophic side compared to the healthy side measured by HFU before and after each lipofilling session.

Case number/sex/age	Diagnosis	Number of LF sessions	Location of LF	Atrophic skin thickness before LF	Atrophic skin thickness after 1st LF	Atrophic skin thickness after last LF
1/F/43	Progressive facial hemiatrophy	3	Malar, cheek	79%	90%	104%
2/M/23	Progressive facial hemiatrophy	3	Malar	83%	80%	99%
3/F/58	Dermatomyositis	3	Malar, cheek	35%	97%	100%
4/M/21	Progressive facial hemiatrophy	1	Forehead, Chin	45%	81%	81%
5/F/32	Morphea “en coup de sabre”	2	Forehead	55%	66%	103%
6/F/30	Progressive facial hemiatrophy	1	Chin	57%	86%	86%
7/F/66	Lupus panniculitis	2	Cheek	59%	76%	82%
8/F/45	Lupus panniculitis	1	Cheek	80%	85%	85%
9/F/52	Morphea “en coup de sabre”	3	Forehead, Cheek, Chin	52%	66%	83%
10/F/46	Progressive facial hemiatrophy	4	Forehead, Malar, Cheek	50%	Data not available	70%
11/F/51	Progressive facial hemiatrophy	5	Forehead, Cheek, Chin	48%	Data not available	71%

*Note:* For simplicity, the table shows the average values per patient, combining all anatomical locations. However, statistical analyses were conducted using data from each individual location per patient. Abbreviations: F, female; Num, number; M, male; LF, lipofilling.

**FIGURE 1:**
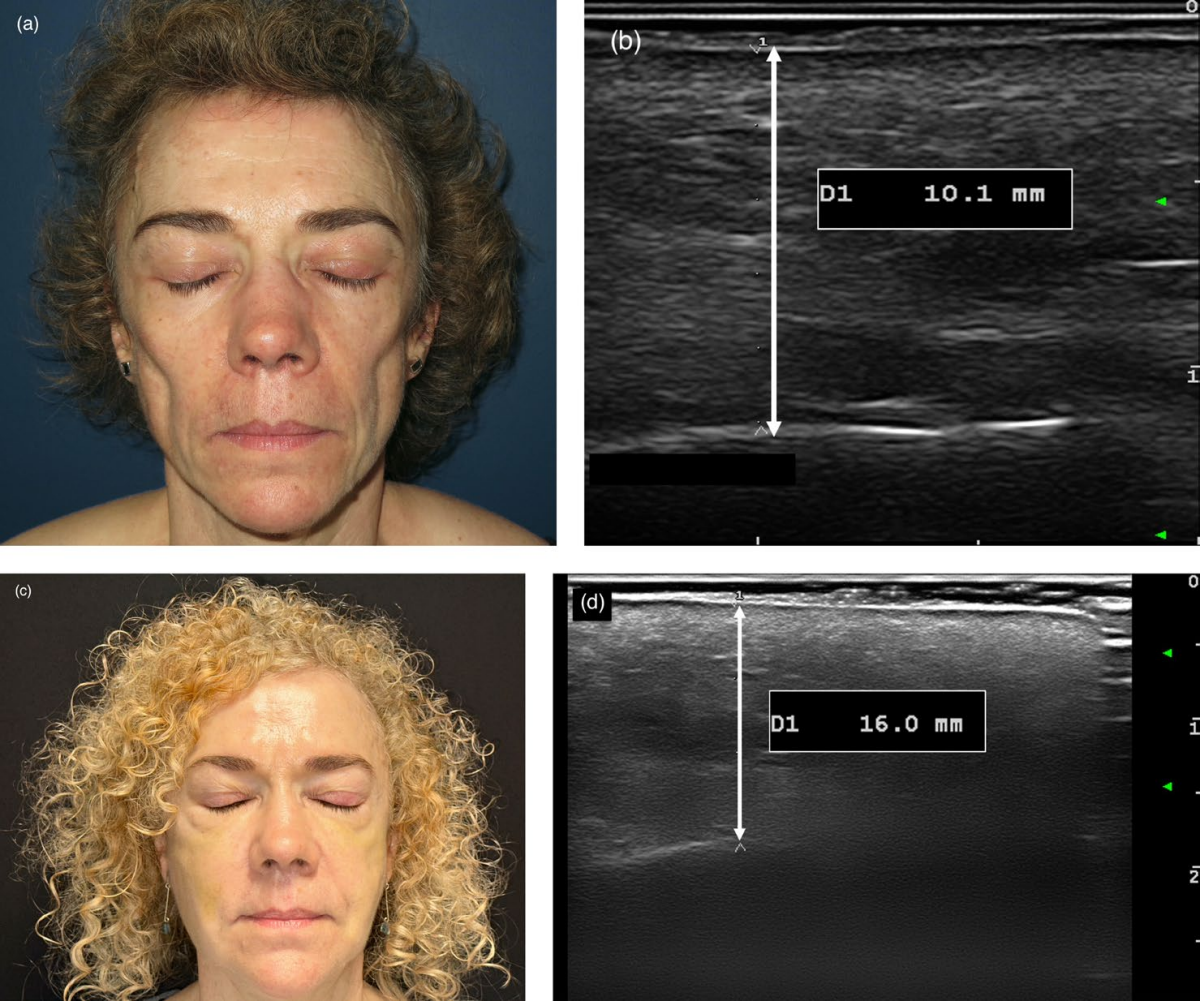
“58‐year‐old female with marked bilateral malar and cheek atrophy due to dermatomyositis.” (a) “Clinical image before lipofilling.” (b) “High‐frequency ultrasound (HFU) measure of skin thickness from the epidermis to the underlying bone in the malar area before lipofilling.” (c) “Clinical image 2 weeks after the 3rd lipofilling session.” (d) “HFU measure of skin thickness in the malar area 2 weeks after the 3rd lipofilling session.”

## Author Contributions

S.G.‐M. and P.G. conceived and designed the study. S.G.‐M. collected the data and performed the statistical analysis. Ultrasound examinations were performed and interpreted by P.G. and M.D.C. Lipofilling procedures were carried out by A.F.F., F.J.C.G., and C.M.P. All authors contributed patients to the case series. The manuscript was written by S.G.‐M. and critically revised by P.G. and J.‐M.M. All authors reviewed and approved the final version of the manuscript.

## Ethics Statement

The patients in this manuscript have given written informed consent to the publication of their case details.

## Conflicts of Interest

The authors declare no conflicts of interest.

## Data Availability

The data that support the findings of this study are available from the corresponding author upon reasonable request.
